# Efficacy and safety of prolonged-release hyaluronic acid derivative vaginal application in the postpartum period: a prospective randomised clinical trial

**DOI:** 10.1080/07853890.2021.1974083

**Published:** 2021-09-03

**Authors:** Claudio Gustavino, Paolo Sala, Nadia Cusini, Brunella Gravina, Cecilia Ronzini, Diletta Marcolin, Valerio Gaetano Vellone, Michele Paudice, Rossella Nappi, Sergio Costantini, Simone Ferrero, Fabio Barra

**Affiliations:** a Unit of Obstetrics and Gynecology, IRCCS Ospedale Policlinico San Martino, Genoa, Italy; b Academic Unit of Obstetrics and Gynecology, IRCCS Ospedale Policlinico San Martino, Genoa, Italy; c Department of Neurosciences, Rehabilitation, Ophthalmology, Genetics, Maternal and Child Health (DiNOGMI), University of Genoa, Genoa, Italy; d Department of Surgical and Diagnostic Sciences, IRCCS Ospedale Policlinico San Martino, University of Genova, Genoa, Italy; e Research Center for Reproductive Medicine, Gynecological Endocrinology and Menopause, IRCCS S. Matteo Foundation, Pavia, Italy

**Keywords:** Prolonged-release hyaluronic acid derivative vaginal gel, hydeal-D, sexual function, postnatal depression, vaginal maturation index, vaginal dryness, vaginal lubrification, randomised trial, postnatal depression, vaginal pH

## Abstract

**Introduction:**

In puerperium, the hypoestrogenic state induced by delivery and subsequently sustained by lactation may lead to vaginal dryness, burning, and itching sensation, contributing to the onset of sexual dysfunction.

**Material and methods:**

This was a prospective, randomized, controlled, open-label study (NCT04560283) for evaluating the effects of application of a prolonged-release hyaluronic acid derivative vaginal gel in restoring sexual function during the postpartum period. Eighty-five patients were randomized to apply prolonged-release Hydeal-D 0.2% vaginal gel (Fidia Farmaceutici, Abano Terme, Italy; *n* = 43) every three days for 12 consecutive weeks or expectant management (*n* = 42).

**Results:**

Women undergoing treatment had a more elevate increase in Female Sexual Function Index (FSFI) total score (+15.1 ± 11.9 vs +6.5 ± 8.9, *p* < 0.001) and a higher decrease in vaginal pH (−1.2 ± 0.7 vs −0.2 ± 1.1; *p* < 0.001). Moreover, the proportion of vaginal smears with maturation index (VMI) >65 was significantly higher in patients treated (80.6% vs 35.3%; *p* = 0.004). Edinburgh Postnatal Depression Scale (EPDS) decreased significantly in both groups with no inter-group difference (*p* = 0.459). Only two cases (4.8%) of moderate vaginal burning sensation were reported in patients undergoing local vaginal therapy.

**Conclusions:**

The results of our study demonstrated that hyaluronic acid derivative vaginal gel (Hydeal-D) was able to improve sexual function of puerperal women in the short-term treatment.

**KEY MESSAGE**
- In the puerperium, the hypoestrogenic state induced by delivery and subsequently sustained by lactation may lead to vaginal dryness, burning, and itching sensation, contributing to the onset of sexual dysfunction.
- Hydeal-D is a prolonged-release hyaluronic acid derivative characterised by elevated resistance to enzymatic breakdown. During puerperium, its local application may improve the vaginal microenvironment by ensuring a better migration and proliferation of cells involved in local tissue repair.
- Among puerperal women, Hydeal-D vaginal gel causes a significant improvement of sexual function, including desire, arousal, and lubrification, compared to expectant management. Furthermore, it leads to a decrease in vaginal pH and an increase of the trophic status of vaginal epithelium.

## Introduction

Sexual dysfunction is a common problem affecting 40–80% of women during the first months after delivery [1]. Despite the critical role of psycho-social factors occurring after childbirth, vaginal dryness and perineal pain are essential contributors to postpartum sexual dysfunction [2].

In puerperium, vaginal hydration and lubrication may be notably altered, causing considerable discomfort characterised by vaginal dryness, burning, and itching sensation. This is partly due to the hypoestrogenic state following delivery and subsequently sustained by lactation, as elevated prolactin levels decrease the production of ovarian oestrogens [3]. Additionally, vaginal delivery can be complicated by perineal tears due to laceration or episiotomy; these traumatic events may be responsible for prolonged perineal pain [4]. All these factors can lead to a negative impact on women’s sexual function after childbirth.

Hyaluronic acid is an anionic, nonsulfated glycosaminoglycan with a relevant function in extracellular matrix, diffuse in the skin, the vaginal mucosa, and several other epithelial tissues. Hyaluronic acid, having hydrating properties, significantly contributes to viscoelastic tissue structure [5]. Additionally, it is involved in several wound-healing mechanisms by inducing cell proliferation and favouring angiogenesis [6,7]. It has been suggested that the presence of hyaluronic acid may be involved in epithelial response to tissue injury after childbirth, stimulating a rapid modification of the cervical and vaginal matrix back to the nonpregnant state [8]. Previous findings suggested that the use of hyaluronic acid exerts effects similar to vaginal oestrogens when relieving vaginal atrophy associated symptoms in postmenopausal women [9]; however, until now, the application of hyaluronic acid during puerperium has never been evaluated.

Hydeal-D is a prolonged-release hyaluronic acid derivative [10]. Being in contact with the vaginal mucosa esterase, this product gradually releases hyaluronic acid by hydrolysis of ester bonds [11]. Previously, this compound has been investigated in postmenopausal women, showing a benefit in the treatment of symptoms, such as vaginal dryness, itching, dyspareunia, related to vaginal atrophy [9,11].

The rationale of its local use during puerperium is to improve the vaginal microenvironment allowing for a better migration and proliferation of cells involved in local tissue repair. Additionally, Hydeal-D may guarantee a durable hydrating action due to persistent adhesion to the mucosa, favouring the spontaneous healing of the microlesions caused by friction due to vaginal dryness.

This randomised, controlled study aims to evaluate efficacy and safety of the prolonged-release hyaluronic acid derivative Hydeal-D vaginal gel during the postpartum period with a special focus on sexual function.

## Material and methods

This prospective, single-center, randomised, controlled, open-label study was performed in an Italian academic hospital (UO Ostetricia e Ginecologia, IRCCS Ospedale Policlinico San Martino, Genova, Italy). Data were collected from November 2016 to April 2020.

The primary objective of this study was to investigate the change in sexual function in puerperal women undergoing vaginal application of the prolonged-release hyaluronic acid derivative vaginal gel. Secondary objectives of this study were: (1) change in vaginal pH and trophism of the vaginal epithelium; (2) evaluation of presence and intensity of symptoms related to postnatal depression; (3) treatment tolerability.

After delivery (V0), women were recruited at the hospital discharge, screened for eligibility criteria, and were advised not to apply any vaginal product to improve vaginal dryness and vaginal perineal healing for the following 40 days. Exclusion criteria for eligibility were allergy to hyaluronic acid gel, signs of vaginal infection, previous of diagnosis of gynecological cancers, recent genital bleeding of unknown, acute hepatopathy, embolic disorders, severe primary disease of the kidney, and mental disorders diagnosed before the previous pregnancy.

At 40th day postpartum (V1), women underwent randomisation with a 1:1 ratio to (1) application of Hydeal-D 0.2% vaginal gel (HYALOGYN^®^; Fidia Farmaceutici, Abano Terme, Italy) every 3 days up to a total of 12 consecutive weeks (group H); (2) expectant management, which consisted in no application of any vaginal product, allowing only daily intimate cleansing (group E). After 12 weeks, patients in both groups underwent follow-up visit (V2).

Hydeal-D is a prolonged-release hyaluronic acid derivative characterised by elevated resistance to enzymatic breakdown. The carbomer and propylene glycol, combined with the hyaluronic acid derivative, enable it to achieve a thick, viscous form [10].

Randomisation was done at V1 using a web-based randomisation application; randomisation lists were made adopting Moses’s algorithm to minimise study bias while maintaining treatment balance throughout the trial. The investigators were able to randomise patients to the study treatments using individual protect log granted access to a study-dedicated randomisation web page.

At V1 and V2, sexual function and perineal pain were evaluated. Sexual function was assessed by the Female Sexual Function Index (FSFI), previously adopted also for evaluation women in the puerperium [12]. The FSFI questionnaire contains 19 questions grouped into six domains: (1) sexual desire; (2) sexual excitement; (3) need for lubrication;(4) orgasm achievement; (5)general sexual satisfaction; (6) pain during sex. All questions are formatted using a multiple-choice system. A score of 0–5 is assigned to each response, and a final sexual satisfaction value is derived mathematically, with the ultimate sexual function score ranging from 2 to 36. Perineal pain was subjectively evaluated using a 10-point visual analogue scale (VAS; 1 = absence of pain; 10 = pain with the highest intensity).

**Figure 1.**
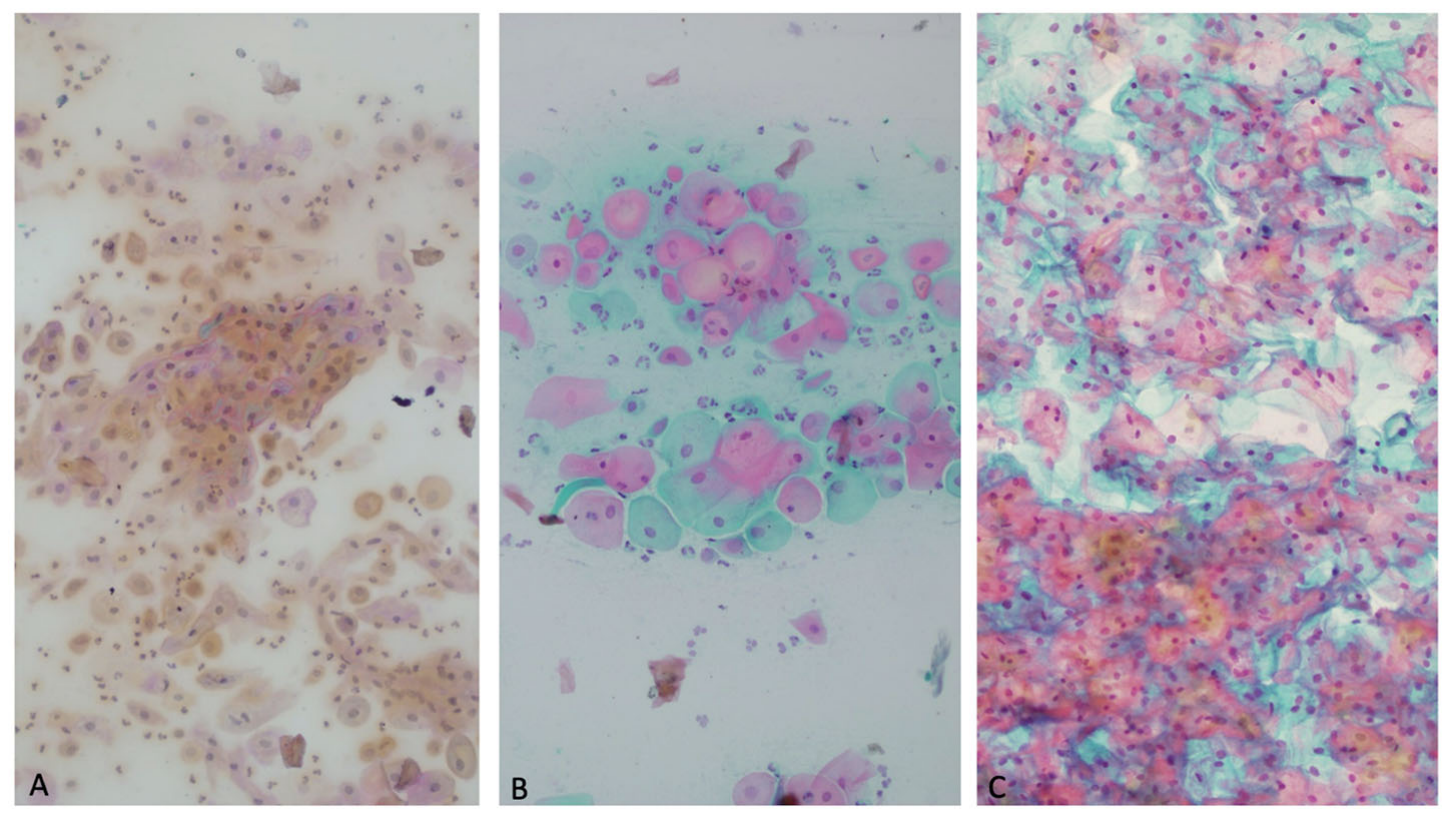
Cytological appearance of vaginal smear. Low vaginal maturation index (A); 50–64 moderate vaginal maturation index (B); high vaginal maturation index (C).

At the same follow-up visits, patients underwent vaginal pH and vaginal maturation index (VMI) assessment. Vaginal pH was measured by a vaginal dipstick (pH 4.0–7.0; Merck KGaA, Darmstadt, Germany). VMI was calculated, according to the formula: maturation value = (0 × % of parabasal cells) + (0.5× % of intermediate cells) + (1.0× % of superficial cells) [13]. As previously reported [14,15], VMI ranging from 0 to 49 indicates low oestrogen effect, 50 to 64 indicates moderate oestrogen effect, and 65 to 100 indicates high oestrogen effect on the vaginal epithelium (Figure 1).

Edinburgh Postnatal Depression Scale (EPDS) was employed for investigating the presence of symptoms suggestive of postnatal depression. EPDS, previously described and validated in the literature [16,17], is composed of 10 items with four possible answers, scoring from 0 (absence of the symptom) to 3 (symptom very intense and present for most of the time). The EPDS total score is derived mathematically, ranging from 0 (no signs of postnatal depression) to 30 (high-intensity symptoms of postnatal depression).

The number and percentage of adverse events (AEs) and the relative CI 95% (exact Clopper Pearson CI) were collected in the two study groups.

## Ethical approval

The study was carried out in compliance with the ethical principles of the Declaration of Helsinki, the Good Clinical Practices International Conference on Harmonisation Guidelines, and the ISO14155 Clinical investigation of medical devices for human subjects – Good Clinical Practice. The study was approved by the local ethics committees (Comitato Etico Regione Liguria; prot. HYDEAL-D-1-2016; Approval 02-03-2016). All subjects provided signed informed consent. The trial was registered on www.clinicaltrials.gov (identifier: NCT04560283).

## Statistical analysis

Study data were summarised using descriptive statistics: categorical variables in terms of frequencies and percentage, continuous variables of mean ± standard deviation (SD), median, interquartile range quartile (IRQ), and min-max value. Shapiro Wilk test and graphical methods were used to evaluate normality assumptions.

Assuming a moderate within-patient correlation between baseline and 12 weeks FSFI scores, that is *ρ* = 0.7 (implying that less than 50% of the total variance in 12 weeks scores is explained by differences in baseline scores) the correction factor (1−*ρ*2) = 0.51, and the sample size required for detecting with power = 80% and alfa = 0.05 a difference in FSFI equivalent to less than 1⁄2 of the standard deviation (primary endpoint), after adjustment for baseline scores, is 31 patients per arm (total = 62). Accounting for a drop-out rate of 10% (i.e. patients not completing all the study visits), the minimum number of women to enrol was at least 33 patients per arm.

At V1, distribution of vagina pH classes and VMI was reported by group. Change in vaginal classes was evaluated in each group separately by Wilcoxon paired test. Differences in changes between groups were assessed by Mann Whitney test on differences between V2 and V1. Change in VAS was evaluated in each group separately by Wilcoxon paired test. Change in FSFI subscale score and EPDS at V2 was calculated as the difference between V2 and V1, and the nonparametric Mann Whitney test was used to compare changes in the two groups. A *p*-value <.05 was considered statistically significant for all statistical tests.

## Results

**Figure 2.**
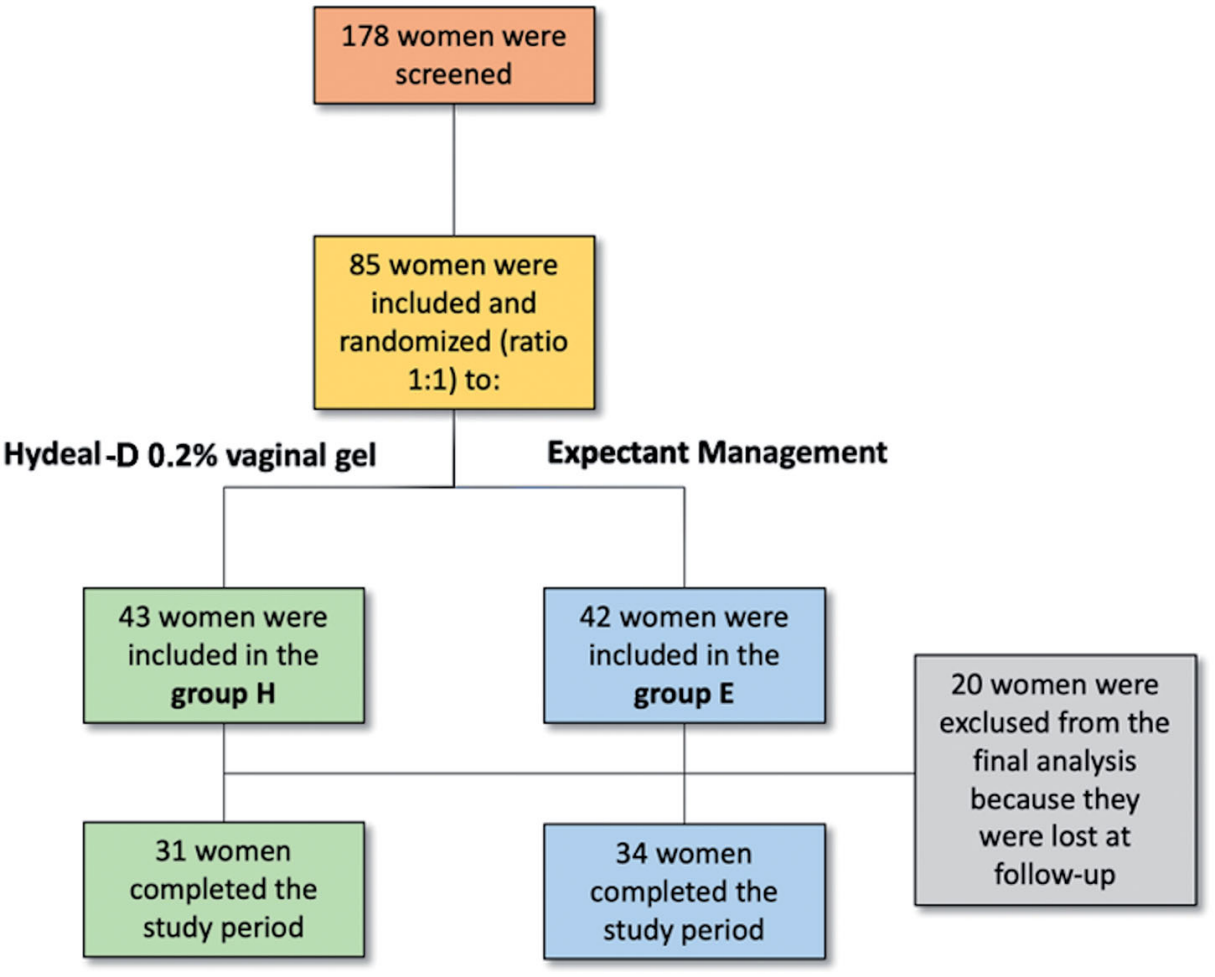
Flow-chart of the study.

One hundred seventy-eight patients were screened in the study; among them, 85 were randomised in group H (*n* = 42) or group E (*n* = 43); twenty patients were excluded from definitive analysis as they did not complete all the study visits (Figure 2). The mean (±SD) age of patients was 34.1 ± 3.8 and 33.0 ± 4.5 years in group H and E, respectively (*p* = .365). In both groups, the proportion of patients undergoing vaginal delivery, operative vaginal delivery, and delivery by caesarean section was similar (*p* = .100); additionally, a similar number of women underwent breastfeeding and/or formula feeding (*p* = .425). The other demographic characteristics are reported in Table 1.

**Table 1.** Demographic characteristics of the study population.

	Group H (*n* = 31)	Group E (*n* = 34)	*p* Value
Age Mean ± SD	34.1 ± 3.8	33.0 ± 4.5	.365
Civil status %, *n*			
*Unmarried*	6.5% (2)	5.9% (2)	.658
*Married*	54.8% (17)	64.7% (22)	.211
*Separate*	0.0% (0)	0.0% (0)	–
*Cohabiting*	38.7% (12)	29.4% (10)	.298
*Widow*	0.0% (0)	0.0% (0)	–
Smoking habits %, *n*			
*Smoker*	3.2% (1)	14.7% (5)	.121
* Ex-smoker*	19.4% (6)	2.9% (1)	.06
* Never smoked*	74.2% (23)	79.4% (27)	.419
*Unknow*	3.2% (1)	2.9% (1)	.730
Alcohol consumption %, *n*			
*Never*	35.5% (11)	52.9% (18)	.122
*Occasionally*	64.5% (20)	44.1% (15)	.081
*Daily*	0.0% (0)	0.0% (0)	–
*Unknow*	0.0% (0)	2.9% (1)	.523
Previous delivery %, *n*	38.7% (12)	32.4% (11)	.198
Delivery type %, *n*			
*Vaginal*	64.5% (20)	41.2% (17)	.575
* Operative vaginal*	9.7% (3)	5.9% (2)	.455
*Caesarean section*	25.8% (8)	52.9% (15)	.100
Vaginal tears* %, *n*	20 (87.0%)	15 (78.9%)	.173
* I grade*	6 (30.0%)	5 (33.3%)	.711
II grade	14 (60.0%)	9 (60.0%)	.882
III grade	0 (0)	1 (6.6%)	.443
IV grade	0 (0)	0 (0)	–
Feeding type %, *n*			
Breastfeeding	58.1% (18)	55.9% (19)	.425
Formula feeding	9.7% (3)	14.7% (5)	.408
Both	32.3% (10)	29.4% (10)	.508

*Evaluated in patients undergoing vaginal and operative vaginal delivery.

At baseline, there was no difference in mean (±SD) total FSFI score between group H and group E (9.2 ± 8.3 and 8.6 ± 8.7, respectively; *p* = .906). Moreover, pH was not different (5.3 ± 0.6 and 5.1 ± 0.5, respectively; *p* = .124), and the VMI classes had a similar distribution between the two groups (*p* = .289). Additionally, the patients had a mean superimposable EPDS score (4.8 ± 3.9 and 6.8 ± 4.6, respectively; *p* = .112).

**Figure 3.**
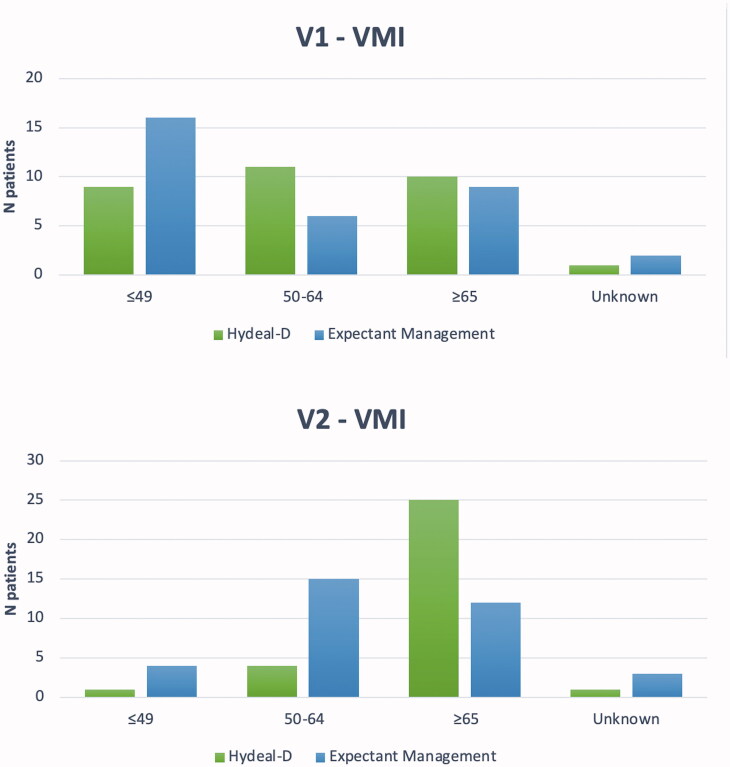
Vaginal maturation index at V1 and V2.

At V2, sexual activity was restarted in 96.8% (*n* = 30/31) and 94.1% (*n* = 32/34) of patients in group H and E. In group H, there was a higher increase in FSFI total score (+15.1 ± 11.9 and +6.5 ± 8.9, respectively; *p* < .001); in particular, the improvement was significant in all the FSFI subdomains (Table 2). Vaginal pH decreased more in women in group H (−1.2 ± 0.7 vs. −0.2 ± 1.1, respectively; *p* < .001; Table 3); specifically, at V2 mean pH was 4.1 ± 0.9 and 5.0 ± 1.2 in the groups H and E, respectively. Furthermore, there was a significant difference in VMI between the two groups (*p* = .003); moreover, the proportion of patients with VMI >65 was significantly higher in group H (80.6% vs. 35.3%; 25/31 vs. 12/34, respectively; *p* = .004; Figure 3). In group H, there was a higher decrease of VAS (−29.0 ± 23.5 vs. +4.7 ± 11.9, respectively; *p* = .008) with a mean VAS value of 7.8 (± 15.8) and 29.8 (± 20.6) for the group H and E, respectively (*p* < .001), at the end of the study. EPDS decreased significantly in both groups (*p* < .001 and *p* = .002, respectively), with no inter-group difference (*p* = .459; Table 3).

**Table 2.** Change in total Female Sexual Function Index (FSFI) and FSFI subdomains between V1 and V2.

Change	Summary statistics	Group H (*n* = 31)	Group E (*n* = 34)	*p* Value
*Desire*	Mean ± SD	1.28 ± 0.96	0.21 ± 1.16	<.001
	Median (IQR)	1.20 (0.6; 1.8)	0.00 ( −0.3; 1.2)	
	Min-Max	−0.6; 3.0	−3.0; 2.4	
*Arousal*	Mean ± SD	3.2 ± 1.8	1.8 ± 2.0	.005
	Median (IQR)	3.9 (2; 5)	2.3 (0; 4)	
	Min-Max	−0; 6	−2; 5	
*Lubrication*	Mean ± SD	4.2 ± 1.6	1.6 ± 1.7	<.001
	Median (IQR)	4.8 (4; 5)	2.1 (0; 3)	
	Min-Max	0; 6	−2; 5	
*Orgasm*	Mean ± SD	3.9 ± 1.5	1.6 ± 1.7	<.001
	Median (IQR)	4.4 (4; 5)	2.0 (0; 3)	
	Min-Max	0; 5	−2; 6	
*Satisfaction*	Mean ± SD	3.7 ± 1.8	1.7 ± 1.8	<.001
	Median (IQR)	4.8 (3; 5)	2.4 (0; 3)	
	Min-Max	−1; 6	−2; 5	
*Pain*	Mean ± SD	4.4 ± 2.1	1.8 ± 2.0	<.001
	Median (IQR)	4.8 (3; 6)	2.8 (0; 3)	
	Min-Max	0; 6	−2; 5	
**FSFI total score**	Mean ± SD	20.43 ± 8.89	8.78 ± 9.30	<.001
	Median (IQR)	22.30 (15.8; 27.1)	11.60 (−0.2; 16.4)	
	Min-Max	−0.5; 30.6	−9.4; 21.4	

**Table 3.** Change in vaginal PH and EPDS between V1 and V2.

Change	Summary statistics	Group H (*n* = 31)	Group E (*n* = 34)	*p* Value
*Vagina pH*	Mean ± SD	−1.2 ± 0.7	−0.2 ± 1.1	<.001
	Median (IQR)	−1.5 (−2; −1)	−0.2 (−1; 1)	
	Min-Max	−3; 1	−4; 2	
*EPDS*	Mean ± SD	−2.8 ± 4.1	−2.2 ± 3.8	.459
	Median (IQR)	−2.0 (−6; 0)	−1.5 (−4; 0)	
	Min-Max	−11; 6	−12; 4	

All the patients in group H did not experience difficulty applying the vaginal gel; in almost all the cases, patients had high compliance to the local treatment (96.8%; 30/31). Two women reported moderate vaginal burning sensation (4.8% CI 95% 0.6–16.2%), nevertheless not causing discontinuation from the local treatment; serious adverse events were not observed in both groups.

## Discussion

This is the first study evaluating the application of prolonged-release hyaluronic acid derivative (Hydeal-D) during puerperium. Previously, hyaluronic acid has been investigated in postmenopausal women, showing a benefit in the treatment of symptoms, such as vaginal dryness, itching, dyspareunia, related to vaginal atrophy [9]. The prolonged-release hyaluronic acid derivative Hydeal-D has been previously employed to reduce vaginal dryness in post-menopause, demonstrating to be a valid alternative to local oestrogen [11]; moreover, it has been successfully investigated to prevent sexual dysfunction in patients assuming adjuvant hormonal therapy for treating breast cancer [18].

Our study demonstrated that in the postpartum period prolonged-release hyaluronic acid derivative vaginal gel improved sexual function after three months of treatment. Of note, women undergoing hyaluronic derivate application had significant improvement in all domains of the sexual function evaluated by FSFI (mean total FSFI score increase: +15.1 vs. +6.5, respectively; *p* < .001), including desire, arousal, lubrification, orgasm, satisfaction, and pain (Table 2). Furthermore, in our study, vaginal pH decreased after local therapy with values reaching physiological vaginal ranges (<5) of no pregnant women (*p* < .001; Table 3) [19]; lastly, the proportion of patients with VMI >65 was significantly higher among patients treated (*p* = .004). Otherwise, our findings did not reveal differences for mean EPDS total score between patients treated and those who underwent expectant management (*p* = .459; Table 3). It is likely that a better recovery of sexual function has a limited impact on this outcome, or alternatively the sample was underpowered to capture the impact on postnatal depression.

The vaginal epithelium is a critical barrier to microbial infection and environmental irritants [20]. During pregnancy, the placenta produces a high amount of oestrogens [21]. Their serum increased levels favour the presence of *Lactobacillus spp.* in vagina through oestrogen-driven maturation of the vaginal epithelium that leads to the accumulation of glycogen. Sexual function is significantly low after delivery because of multiple biological factors: the rapid reduction of oestrogen levels in the postpartum period causes a relevant decrease in vaginal glycogen; glycogen break down products utilised by lactate-producing bacteria, subsequently reducing the community stability and resilience of vaginal microbiome, and in particular, of *Lactobacillus* spp. These events tend to cause an increase of vaginal pH, favouring microbial imbalance and infection [22]. Additionally, complications occurring during pregnancy, delivery, and postpartum, like genital infections, haemorrhage, may influence the onset of subsequent sexual function [1]. Diminished vaginal hydration and lubrication due to the increased pH and decreased tissue trophism for the lack of oestrogens may contribute to the onset of sexual dysfunction [2]. The presence of transient vaginal atrophy in 215 puerperal women has been only investigated in a dated trial with the following criteria defining a vaginal atrophic status : vaginal pH ≥5.3 and/or at least one of vaginal symptom/sign [23]. At a 4-week postpartum visit, thirty-seven patients showed vaginal atrophy (17.2%) with a mean pH of 6.55; the mean VMI was 27.5 (49.2% parabasal, 46.5% intermediate, and 4.3% superficial cells). In this group of patients, eight out of 10 patients who attempted coitus complained of dyspareunia (80%), 6 (16.2%) had vaginal stinging, 4 (10.8%) had vaginal tightness, and 1 (3%) had dysuria. Four women (10.8%) had experienced similar symptoms after previous pregnancies. Eighty patients (37.2%) did not reach inclusion criteria for vaginal atrophy, having a mean VMI of 62.9 (0.3% parabasal, 73.6% intermediate, and 26.1% superficial cells) and a pH of 4.68. Notably, this study established a statistically significant (*p* < .0001) relationship between vaginal pH and maturation index.

It has been previously shown that vaginal delivery and caesarean section do not significantly influence the resumption of sexual intercourse [24]. Nevertheless, breastfeeding may alter sexual function due to vaginal dryness produced by the high levels of prolactin and lowered oestrogen levels. Indeed, the elevated prolactin levels tend to suppress the pulsatile secretion of gonadotropin-releasing hormone, maintaining a degree of constant gonadal suppression [3]. In our study, the proportions of women who underwent caesarean section and breastfeeding were similar in the two study groups (*p* = .100 and *p* = .425, respectively), allowing us to study the effect of the therapy independently from these potential influencing factors.

Previous authors demonstrated on the animal model that high collagen concentration causes an increase in remodelling process by enhancing production of hyaluronic acid in vagina and its supporting tissues [25]. The local application of hyaluronic acid vaginal gel does not negatively impact the microenvironment of vagina, not having a physio-chemical irritating action; additionally, hyaluronic acid is characterised by a high safety profile, which allows for an elevated patients’ compliance to the treatment [11]. In our study, during the treatment, only two women experienced a vaginal burning sensation of moderate intensity (4.8%), which nevertheless did not cause discontinuation from therapy. These data confirmed the high tolerability of local vaginal application of hyaluronic acid.

Production of hyaluronic acid by fibroblasts during the proliferative stage of wound healing stimulates the migration and mitosis of fibroblasts and epithelial cells [6]. For this reason, the use of prolonged-release hyaluronic acid is supposed to contribute to tissue regeneration and healing process, particularly relevant after the traumatic events related to vaginal delivery. Moreover, it favours repair of vaginal epithelial injuries, inducing the expression of vascular endothelial growth factor (VEGF)-driven angiogenesis and at the same time hydrating the vaginal mucosa; this ultimately helps the vaginal tissue to regain elasticity and softness [26]. In our study, almost all the patients undergoing vaginal delivery had I-II grade perineal tears ; therefore, it was not possible to draw conclusion on the use of hyaluronic acid specifically on patients with higher-grade (III-IV) vaginal tears. Although prolonged-release hyaluronic acid acts on vaginal epithelium, involved in all the grades of vaginal tears , it would be of interest to investigate if its use may further favour the vaginal repair of this subpopulation of patients.

It has previously reported that maintaining adequate or improving the state of vaginal health and acting positively on the cervical epithelium and the vaginal microbiota could be a new strategy to prevent both the acquisition of sexually transmitted infection [27,28]. Hyaluronic acid induces vaginal epithelial cells to release different antimicrobial peptides, such as defensins mediated by toll-like receptor (TLR)-2 and TLR-4 [29]. The innate host defense has a critical role during injury restitution. Vaginal surface acts as efficient player of innate host defense, which may modulate its antimicrobial properties and injury restitution activity, following hyaluronic acid stimulation [30]. Overall, the biological activity of prolonged-release hyaluronic acid derivative may give an additional protective action for the vaginal epithelium, which is highly and constantly exposed to microbiota, thereby facilitating the self-defence of the vaginal epithelium [20].

The strengths of this study are represented by the study design, which was based on randomisation and control arm, and included a standardised evaluation of sexual function by FSFI. Another advantage is that the sample size has been determined a priori by performing a power calculation. Limitations of this study were the facts that treatment was not blinded, for either the investigator or the patient and that the statistical analysis was not performed by a blind statistician. Additionally, vaginal pH and vaginal maturation index, employed for evaluating secondary study outcomes,are indirect indicators of vaginal trophic status. In the near future, it would be of interest to further investigate the impact of prolonged-release hyaluronic acid derivative on histology obtained from vaginal biopsy, which was not planned in the present study to increase the patients’ participation to this study investigating the impact of such local therapy in the puerperal vaginal tissue.

## Conclusions

The prolonged-release hyaluronic acid derivative (Hydeal-D) vaginal gel may improve sexual function during three months of treatment in postpartum period. The present findings revealed that this local therapy compared to expectant management causes a significant improvement in several aspects of sexual function, including desire, arousal, lubrification, orgasm, satisfaction, and pain. Furthermore, it leads to a decrease in vaginal pH and an improvement of the trophic status of vaginal epithelium.

## Data Availability

The data that support the findings of this study are available from the corresponding author, SF, upon reasonable request.
